# Educational Attainment Inequalities in Depressive Symptoms in More Than 100 000 Individuals in Europe

**DOI:** 10.1192/j.eurpsy.2022.689

**Published:** 2022-09-01

**Authors:** A. Chlapecka, A. Kagstrom, P. Cermakova

**Affiliations:** 1 Third Faculty of Medicine, Charles University in Prague, Department Of Psychiatry And Medical Psychology, Klecany, Czech Republic; 2 Centre of Clinical Neuroscience, Department Of Neurology, Prague, Czech Republic; 3 National Institute of Mental Health, Public Mental Health, Klecany, Czech Republic; 4 2nd Faculty of Medicine, Charles University in Prague, Department Of Epidemiology, Prague, Czech Republic

**Keywords:** education, Depression, Epidemiology, Europe

## Abstract

**Introduction:**

Increasing educational attainment (EA) could decrease the occurrence of depression. We investigated the relationship between EA and depressive symptoms in older individuals across four European regions.

**Objectives:**

1) examine association between EA and depressive symptoms 2) determine, if there is an upper limit to this association 3) explore regional and demographic differences within this relationship across Europe

**Methods:**

We studied 108 315 Europeans (54 % women, median age 63 years old) in Europe assessing EA and depressive symptoms. Logistic regression estimated the association between EA and depressive symptoms, adjusting for sociodemographic and health-related factors; testing for sex/age/region and education interactions.

**Results:**

Higher EA was associated with lower odds of depressive symptoms, independent of sociodemographic and health-related factors. A threshold of the lowest odds of depressive symptoms was detected at the first stage of tertiary education (OR 0.60; 95% CI 0.55-0.65; p<0.001; relative to no education). Central and Eastern Europe showed the strongest association (OR for high vs. low education 0.37; 95% CI 0.33-0.40; p<0.001) and Scandinavia the weakest (OR for high vs. low education 0.69; 95% CI 0.60-0.80; p<0.001). The association was strongest amongst younger individuals. There was a sex and education interaction only within Central and Eastern Europe.

**Conclusions:**

Level of EA is reflected in later-life depressive symptoms, suggesting that supporting individuals in achieving EA, and considering those with lower EA at increased risk for depression, could lead to decreased burden of depression across the life-course. Further educational support in Central and Eastern Europe may decrease the higher burden of depressive symptoms in women.

**Disclosure:**

No significant relationships.

